# A novel image-based method for simultaneous counting of *Lactobacillus* and *Saccharomyces* in mixed culture fermentation

**DOI:** 10.1093/jimb/kuad007

**Published:** 2023-03-22

**Authors:** Cecelia Williamson, Kevin Kennedy, Sayak Bhattacharya, Samir Patel, Jennifer Perry, Jason Bolton, Lewis Brian Perkins, Leo Li-Ying Chan

**Affiliations:** Department of Advanced Technology R&D, Nexcelom from PerkinElmer, Lawrence, MA 01843, USA; Food Science and Human Nutrition, School of Food and Agriculture, University of Maine, Orono, ME 04469, USA; Department of Advanced Technology R&D, Nexcelom from PerkinElmer, Lawrence, MA 01843, USA; Department of Advanced Technology R&D, Nexcelom from PerkinElmer, Lawrence, MA 01843, USA; Food Science and Human Nutrition, School of Food and Agriculture, University of Maine, Orono, ME 04469, USA; Cooperative Extension, School of Food and Agriculture, University of Maine, Orono, ME 04469, USA; Innovation Program, Maine Busines School, University of Maine, Orono, ME 04469, USA; Food Science and Human Nutrition, School of Food and Agriculture, University of Maine, Orono, ME 04469, USA; Department of Advanced Technology R&D, Nexcelom from PerkinElmer, Lawrence, MA 01843, USA

**Keywords:** *Lactobacillus plantarum*, *Saccharomyces cerevisiae*, Cell counting, Mixed culture, Fermentation, Image cytometry, Cellometer X2

## Abstract

Mixed microorganism cultures are prevalent in the food industry. A variety of microbiological mixtures have been used in these unique fermenting processes to create distinctive flavor profiles and potential health benefits. Mixed cultures are typically not well characterized, which may be due to the lack of simple measurement tools. Image-based cytometry systems have been employed to automatically count bacteria or yeast cells. In this work, we aim to develop a novel image cytometry method to distinguish and enumerate mixed cultures of yeast and bacteria in beer products. Cellometer X2 from Nexcelom was used to count of *Lactobacillus plantarum* and *Saccharomyces cerevisiae* in mixed cultures using fluorescent dyes and size exclusion image analysis algorithm. Three experiments were performed for validation. (1) Yeast and bacteria monoculture titration, (2) mixed culture with various ratios, and (3) monitoring a Berliner Weisse mixed culture fermentation. All experiments were validated by comparing to manual counting of yeast and bacteria colony formation. They were highly comparable with ANOVA analysis showing p-value > 0.05. Overall, the novel image cytometry method was able to distinguish and count mixed cultures consistently and accurately, which may provide better characterization of mixed culture brewing applications and produce higher quality products.

## Graphical Abstract

**Graphical Abstract:**
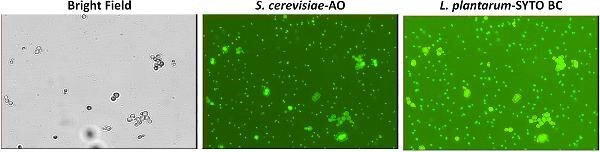
Simple and rapid cell counting method for counting *Lactobacillus plantarum* and *Saccharomyces cerevisiae* mixed cultures using image cytometry.

## Introduction

Mixed cultures have long been a staple in food preparation and have been documented as early as 10 000 BC (Bourdichon et al., 2012; Prajapati & Nair, 2003). Before the advent of modern microbiology, craftsmen relied on spontaneous fermentation to produce tea, beer, cheese, and bread. Many fermentation processes utilize mixed cultures that contain two or more different microorganisms, which may include fungi (e.g., yeast and molds) or bacteria (e.g., lactic acid and acetic acid bacteria) (Hesseltine, 1992a, 1992b). These varieties of mixed cultures are employed for their ability to create unique flavor profiles, health benefits, and food preservation capabilities (Smid & Lacroix, 2013).

Yeasts are microorganisms that are eukaryotic and are members of the fungi kingdom. They are found naturally in the environment and are common on fruit skins, plant surfaces, and attached to some insects (Spencer & Spencer, 1997). Yeasts are well known for their ability to participate in fermentation, and this process occurs mainly in anaerobic or low-oxygen conditions. Yeast anaerobic fermentation yields ethanol and a myriad of other desirable and undesirable metabolic byproducts (Boulton & Quain, 2006; Swiegers et al., 2005). Examples of yeast-fermented products cover a broad range, including alcoholic beverages, kombucha, bread, and biofuels (Liszkowska & Berlowska, 2021; Rojas et al., 2022). Lactic acid bacteria (LABs) are a group of bacteria that are known for their probiotic properties and production of unique flavors in foods such as fermented yogurts, dairy products, and vegetables (Mathur et al., 2020). They can participate in homolactic fermentation or heterolactic fermentation. In the context of mixed culture, different LABs will grow at different time points during fermentation and can be affected by levels of tolerance to alcohol, pH, and the presence of various metabolites. Specifically, *Lactiplantibacillus plantarum* (formerly *Lactobacillus plantarum*) has been studied for its probiotic activity, demonstrating adhesion to gastrointestinal cells, enabling fermentation of silage, and producing antimicrobial substances such as plantarcins that can inactivate pathogens (Soundharrajan et al., 2019). In addition, *L. plantarum* can produce large amounts of β-galactosidase that enable improved lactose digestion (Cebeci & Gürakan, 2003). Furthermore, *L. plantarum* can ferment fructooligosaccharides, which are indigestible sugars that cause dehydration (Cebeci & Gürakan, 2003).

Craft breweries have grown significantly in the United States in the past decade (Brewers Association Releases Annual Craft Brewing Industry Production Report for 2020, 2021). According to the Master Brewers Association, craft brewing reached an astounding 22.2-billion-dollar market in 2020 with ∼9000 operating breweries, showing an annual increase of 4.5% in total operations (Brewers Association Releases Annual Craft Brewing Industry Production Report for 2020, 2021). The growth of demand also came with an appetite for new and unique flavors such as sour beers. Sour beers, like kettle sours, often utilize mixed cultures of yeast and bacteria to create a sweet and sour flavor profile (Hodgkin et al., 2020). Although less popular than modern light beers (pilsners) and a variety of craft beers, sour beers have a much longer history. Modern sour beers can be created with pitched mixed cultures, stepwise fermentation with different microorganisms, or can be brewed using wild fermentation. Sour beers have risen in popularity since the mid-1990s, when only a few craft brewers produced this style. In 2002, The Great American Beer Festival introduced a ‘‘Sour Beer’’ category that had only 15 entries. By 2013, this category boasted 238 entries (Tonsmeire, 2014). Sour beers now comprise ∼11.0% of beer sales and enjoyed a 73% increase in sales growth in 2016 in the United States (Statista, 2022). With the growth of sour beers have come different beer style categories and subsets of those categories. There are approximately eight categories of sour beers, including American wild ale, Berliner Weisse, Flanders red ale, gose, lambic, and Oud Bruin (Tonsmeire, 2014), as well as countless variations within these established categories.

Traditionally, yeast and bacteria can be counted separately using the colony formation assay with the microorganisms streaked onto agar dishes, incubated for several days, and then counted to enumerate colony-forming units (CFUs; Sanders, 2012). However, this method can be time-consuming, requiring 24–72 hr for colony growth and can have high operator-dependent variation. Furthermore, enumeration of a mixed culture would require the use of multiple types of cultural media needing different incubation times, further increasing complexity. Other rapid methods, such as flow cytometry, may be used, but involve substantial capital cost, can be quite expensive to maintain, require a dedicated operator, and may be time-consuming due to the need to label to distinguish the microorganisms (Thomas et al., 2012).

In the last decade, image cytometry has been used in many craft breweries for production and quality control to directly count yeast and measure viability to ensure consistency in beverage products (Chan et al., 2011, 2012; Saldi et al., 2014). Previous publications have also shown accurate direct counting of *Brettanomyces* yeast and *Lactobacillus* bacteria (Hodgkin et al., 2020; Martyniak et al., 2017). In this work, we demonstrate the use of the Cellometer X2 image cytometer to simultaneously count a mixed culture of *Saccharomyces cerevisiae* (*S. cerevisiae*) and *Lactiplantibacillus plantarum* (*L. plantarum*), which has not been shown previously. First, we demonstrate the ability to count titrations of monoculture of yeast and bacteria with fluorescent staining. Second, we verify the use of the bacteria counting chambers for yeast counting. Third, we validate the direct counting of yeast and bacteria with various mixture ratios. Finally, we monitor the yeast and bacteria concentration during a standard mixed culture fermentation of a Berliner Weisse-style beer. In addition, manual counting of CFU is conducted concurrently for direct concentration comparison.

Direct cell counting of mixed culture of yeast and bacteria using chamber-based image cytometric analysis has not been published previously. The utilization of two fluorescent dyes for optimizing staining and distinguishing yeast and bacteria cells, as well as the use of thin chamber slides for ensuring optimal focus, are highly innovative. The proposed novel image cytometry method for mixed cultures can rapidly characterize the concentration of yeast and bacteria microorganisms in beverage products during the course of fermentation, which may improve the consistency and quality of the end products.

## Materials and Methods

### *Saccharomyces cerevisiae* Preparation

*Saccharomyces cerevisiae* (Safale S-04) was purchased from Fermentis (Marcq-en-Barœul, France) in dry yeast packets. Dried yeast packets were rehydrated and grown overnight in 50 mL of potato dextrose broth (Difco, BD, Franklin Lakes, NJ). The yeast culture was isolated with streak plating on acidified potato dextrose agar (APDA, Difco) plates in triplicate. Next, the isolated yeast colonies were aseptically transferred to 10 mL of acidified potato dextrose broth and incubated for 24 hr at 30°C.

For each experiment, an isolated yeast colony was aseptically transferred from APDA plates to 50 mL of potato dextrose broth in triplicate and incubated in a water bath shaker for 24 hr at 30°C. Next, ∼10 mL aliquots were collected from each sample and centrifuged (Eppendorf 5430, Framingham, MA) for 5 min at 4000 RPM (1800 × *g*). Subsequently, the pellet was resuspended in 2 mL of 1X PBS (pH 7.4), yielding an approximate yeast concentration of 10^7^ cells/mL.

### *Lactiplantibacillus plantarum* Preparation

*Lactiplantibacillus plantarum* (*Lactobacillus plantarum* ATCC 8014) was sourced from the American Type Culture Collection (ATCC, Manassas, VA). The strain was stored in 1 mL aliquots of DeMann, Rogosa, and Sharpe (MRS) broth (Difco) mixed 50:50 with 80% glycerol at −80°C. After thawing, each aliquot was transferred to 9 mL of MRS broth and incubated for 24 hr at 30°C. Next, the 24-hr growth culture was streaked onto MRS plates and incubated for 1–2 days at 30°C. For each experiment, a single isolated colony from each MRS streak plate was aseptically transferred to a tube containing 9 mL of sterilized MRS broth. The inoculated samples were incubated for 24 hr at 30°C.

### Fluorescent Stain Preparation and Staining Protocol

The acridine orange (AO) and propidium iodide (PI) fluorescent nuclear stains were provided by Nexcelom (Lawrence, MA) and used to stain *S. cerevisiae* (Chan et al., 2011; Saldi et al., 2014). The ViaStain^™^ AO/PI Staining Solution (CS2-0106-5mL) was used for the initial yeast monoculture staining. For yeast monoculture AO/PI staining, the samples were first diluted 1:1 with the yeast dilution buffer (Nexcelom) and then mixed 1:1 with the AO/PI dye for ∼2 min prior to image cytometric analysis.

The SYTO BC fluorescent stain was purchased from Thermo Fisher Scientific (Carlsbad, CA). The working stock of SYTO BC was prepared by diluting 1:100 in deionized (DI) water. The working stock solution was mixed well and stored in the dark for staining at ambient temperature. Stock solutions were freshly prepared for each experiment. For bacteria monoculture staining, the samples were stained 1:1 with the SYTO BC working solution for ∼2 min prior to image cytometric analysis (Hodgkin et al., 2020).

The ViaStain^™^ AO Staining Solution (CS1-0108-5mL) and SYTO BC were used for yeast and bacteria mixed culture staining. The SYTO BC working stock (1:100 in water) was mixed 2:1 with AO and vortexed. The yeast and bacteria mixed culture was stained 1:1 with the SYTO BC/AO mixed stain for ∼2 min. All the fluorescent stains were prepared fresh for each experiment and stored at ambient temperature for the duration of the experiment.

### Cellometer X2 Image Cytometry Method

The Cellometer X2 image cytometer utilizes a bright field and two fluorescent imaging channels: green (VC-535-402) and red (VC-660-502) for cell count, concentration, and viability measurement (Hodgkin et al., 2020; Martyniak et al., 2017; Saldi et al., 2014). The instrument implements a 10X objective, producing a resolution of ∼0.5 μm^2^/pixel.

For the initial yeast counting experiments, both fluorescent channels were used. The VC-535-402 (excitation/emission: 470 nm/535 nm) was used to detect and enumerate AO-stained cells with exposure times between 500 and 1000 ms, while the VC-660-502 (excitation/emission: 540 nm/660 nm) was used to detect and enumerate PI-stained cells with exposure times between 2200 and 2700 ms. Following the AO/PI yeast staining protocol, 5 μL of the stained sample was pipetted into a Nexcelom counting chamber (CHT4-SD025) and inserted into the system. The chamber was immediately checked under the bright field for appropriate yeast morphology and potential contamination. After the chamber was reviewed and focused, the system would acquire bright field and fluorescent images at four different areas in the counting chamber. The images were analyzed automatically in the software to generate cell count, concentration, and viability using the following counting parameters: fluorescence (FL) channel 1, cell diameter (2.0–30.0 μm), roundness (0.00), fluorescent threshold (25.0), decluster Th factor (0.90); fluorescence (FL) channel 2, cell diameter (2.0–30.0 μm), roundness (0.00), fluorescent threshold (20.0), decluster Th factor (0.90).

For the bacteria counting experiments, only the VC-535-402 channel was used with exposure times between 300 and 1500 ms. Following the SYTO BC staining protocol, 5 μL of the stained sample was pipetted into the CHT4-SD025 counting chamber. The inlet and outlet ports were quickly taped with Scotch tape to prevent evaporation and slow cell movement within the chamber. The cells in the taped chamber were allowed to settle for ∼30 s to further minimize movement, and then inserted into the system for image analysis using the following parameters: fluorescence (FL) channel 1: cell diameter (0.7–40.0 μm), roundness (0.00), fluorescent threshold (10.0), decluster Th factor (0.90).

A similar procedure was performed for analyzing bacteria and yeast mixtures with two fluorescent channels, where both channels were set up with VC-535-402 to detect AO and SYTO BC fluorescence. Slightly stricter image analysis parameters were used to improve cell counting for yeast and bacteria stained with AO and SYTO BC, respectively with the following parameters: fluorescence (FL) channel 1––yeast, cell diameter (6.0–50.0 μm), roundness (0.00), fluorescent threshold (8.0), decluster Th factor (0.90); fluorescence (FL) channel 2––bacteria, cell diameter (0.5–5.0 μm), roundness (0.00), fluorescent threshold (10.0), decluster Th factor (0.90).

### Comparison of SD100 and SD025 Cell Counting Chamber for *S. cerevisiae*

Previous publications have demonstrated the validation of yeast counting in Cellometer X2 image cytometer using the CHT4-SD100 cell counting chambers. Since *S. cerevisiae* and *L. plantarum* mixtures are required to use the CHT4-SD025, we performed a direct concentration comparison of yeast counting in both chambers. First, the stock yeast culture was diluted in deionized (DI) H_2_O to 0.25, 0.5, 0.75, and 1 dilution fractions (*n* = 6/dilution). Each dilution was stained with AO/PI and immediately analyzed using the image cytometer for total concentration comparison.

### Independent Measurement of *L. plantarum* and *S. cerevisiae* Titration

An initial titration experiment was performed for both *L. plantarum* and *S. cerevisiae* to demonstrate the counting capability of the image cytometer. After preparing the stock cultures for yeast and bacteria, the yeast sample was diluted with DI H_2_O to 0.1, 0.3, 0.5, 0.7, 0.9, and 1 dilution fractions (*n* = 4/dilution), while the bacteria sample was diluted to 0.1, 0.25, 0.5, 0.75, and 1 dilution fractions (*n* = 6/dilution). The yeast samples were stained with AO/PI, the bacteria samples were stained with SYTO BC, and subsequently imaged and analyzed using the image cytometer to produce cell concentration results. The titration experiment was repeated two more times and validated against the CFU manual counting method (described below).

### *Lactobacillus plantarum* and *S. cerevisiae* Mixed Culture Detection Validation Experiment

To demonstrate the ability of the image cytometer to correctly identify and count yeast and bacteria in the mixed cultures, we performed three separate experiments at various mixture ratios. First, the stock yeast culture was diluted to 0.001, 0.01, 0.1, 0.2, 0.5, and 1 dilution fractions, and mixed with the stock bacteria culture at 1:1 (*n* = 6/dilution). Second, the stock bacteria culture was diluted to the same dilution fractions and mixed with the stock yeast culture at 1:1 (*n* = 6/dilution). Third, the stock yeast and bacteria cultures were mixed at different percentages of yeast/bacteria: 0, 25, 50, 75, and 100% (*n* = 6/dilution). The mixtures from the three experiments were all stained with the AO/SYTO BC dye mixture and immediately analyzed using the image cytometer for total concentration measurement. The mixed culture detection experiment was repeated two more times and validated against the CFU manual counting method.

### CFU Manual Counting Method

The measured cell concentrations of 24-hr yeast culture were compared between image cytometry and manual plate counting. The stock yeast solution was serially diluted in DI H_2_O from 10^−4^ to 10^−6^ dilution factors in duplicate and then spread onto APDA plates in triplicate. The inoculated plates were incubated for 2–3 days at 30°C. The most countable dilution plates were used, and the rest were discarded.

Similarly, the measured cell concentrations of 24-hr *L. plantarum* cultures were also compared between image cytometry and manual plate counting. The bacteria stock cultures for *L. plantarum* were serially diluted with peptone water from 10^−5^ to 10^−7^ dilution factors in triplicate and then spread onto MRS agar plates in duplicate. The inoculated plates were incubated for 1–2 days at 30°C. The most countable dilution plates were used, and the rest were discarded.

### CFU and Image Cytometry Comparison Using ANOVA

Counting results from the image cytometry and plating methods were back-calculated to the starting concentrations of their respective 24-hr growth cultures. The colony counting (CFU/mL) and cell counting (cells/mL) results were first converted to log scale (base 10), and the average results from each experiment were compared between image cytometry and traditional manual counting method using the ANOVA regression in JMP Pro 15.2.0 (466311). A *p*-value of <.05 was considered statistically significant.

### Berliner Wiesse Style Mixed Culture Fermentation Experiment

A sour beer fermentation experiment was designed and conducted with the mixed culture process to demonstrate the ability of the proposed image cytometry method to accurately count the concentrations of *L. plantarum* and *S. cerevisiae* simultaneously in wort media during active fermentation.

All equipment was cleaned, rinsed, and sanitized before each trial. Five Star PBW (Five Star Chemical, Arvada, CO) was used for cleaning, and Five Star Saniclean Low Foam was used to sanitize. A Sabco Brew-Magic™ system (Toledo, OH) was utilized to mash, sparge, and boil the wort, and a Sabco Chill-Wizard plate chiller (Toledo, OH) was used to cool the wort.

To prepare for the mixed culture fermentation, an isolated colony from the *L. plantarum* preparation was aseptically transferred to 100 mL of MRS broth and incubated for 24 hr at 30°C until a concentration of 10^9^ CFU/mL was achieved. Next, an isolated yeast colony from the *S. cerevisiae* preparation streak plates was aseptically transferred to 50 mL of potato dextrose broth and incubated for 24 hr at 30°C. The 24-hr yeast culture was added to a 1.040 specific gravity (SG) malt-dextrose solution and mixed using a stir bar at room temperature for up to 48 hr or until a concentration of at least 10^8^ CFU/mL was achieved.

The Sabco Brew-Magic™ system was used to prepare ∼6 gallons (22.7 L) of wort to be divided into three fermentation vessels. The wort was prepared by mashing 4.33 lb (1.96 kg) of German pilsner malt, 4.33 lb (1.96 kg) of German wheat malt, and 0.66 lb (0.30 kg) of rice hulls into 3 gallons (11.4 L) of water at 71°C. The mash was stirred every 20 min for a total of 60 min during the mashing step. At the end of the 60 min, the mash was allowed to recirculate (Vorloauf) for 10 min or until the liquid was clear. The grain was then rinsed (sparged) with 4 gallons (15.1 L) of water at 85°C. During sparging, 6 gallons (22.7 L) of wort was transferred to a kettle and boiled (kettled) for 60 min. After boiling, a sample was collected to test the pH and SG. The initial pH averaged 5.66, and the average initial SG was 1.0336. The wort was chilled using a Sabco Chill Wizard plate chiller/heat exchanger (Toledo, OH). Next, ∼0.75 gallons (2.8 L) of chilled wort was transferred to each of the three one-gallon (3.8-L) glass fermentation vessels with lids and three-piece airlocks. Each fermentation vessel was incubated between 22 and 24°C. Finally, the wort was inoculated with an estimated 750 million cells/mL of *S. cerevisiae* and ∼10 million cells/mL of *L. plantarum*, which were typical industry standards for a mixed-cultured beer.

Three independent fermentation trials were performed and monitored. The wort was sampled at 0, 3, 6, 9, 12, 24, and 48 hr, where the concentrations of *L. plantarum* and *S. cerevisiae* were analyzed and monitored using the image cytometer. Similarly, plating and manual counting of CFU were performed on the APDA and MRS plates. For image cytometric analysis, 1.5 mL of sample was collected at each time point, diluted 1:10 in DI water, and then stained with the AO/SYTO BC dye mixture prior to image analysis (*n* = 4). Unstained samples were diluted and plated based on the image cytometry results. The *S. cerevisiae* and *L. plantarum* samples were plated using APDA and MRS plates in triplicate, respectively. The agar plates were incubated at 30°C for 48–72 hr and 24–48 hr for *S. cerevisiae* and *L. plantarum*, respectively, and the resulting manual counting concentrations were compared directly to image cytometry. The resulting concentrations (CFU/mL) of the plating cultures and image cytometer (cells/mL) were converted to a log scale (base 10) for comparison (CFU/mL and cells/mL were used interchangeably). The average log(CFU/mL) or log(cells/mL) values for each experiment were compared using ANOVA in JMP Pro 15.2.0 (466 311). A *p*-value of <.05 was considered statistically significant. Finally, other parameters were collected during the fermentation, such as pH, SG, and aroma profile.

## Results and Discussion

### Verification of SD025 Cell Counting Chamber for *S. cerevisiae*

**Fig. 1.**
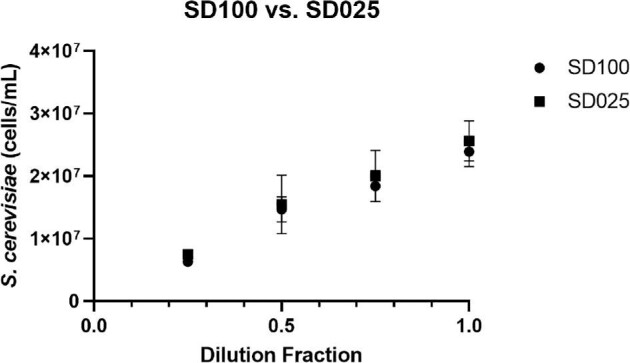
*Saccharomyces cerevisiae* concentration comparison in respect to dilution fractions between SD100 and SD025 counting chambers.

In this experiment, we verified that the use of the SD025 bacteria counting chamber can be used for yeast counting. In previous publications, yeast has been primarily counted in the SD100 chambers, thus it was critical to demonstrate that there are minimal effects on counting yeast in a thinner chamber. The yeast monoculture was first diluted with 0.25, 0.5, 0.75, and 1.00 dilution fractions and then counted with AO/PI staining. The results are shown in Fig. 1, which showed comparable concentration measurements between the two types of consumables. The percentage differences were 17.6, 5.6, 8.3, and 6.7%, respectively, for dilution fractions from 0.25 to 1.00. Overall, a two-sample *t*-test was calculated for each dilution and showed no significant statistical differences (0.66, 0.44, and 0.34) between the two consumables except the 0.25 dilution fraction (0.001), which may be due to cell counting precision at low concentration.

### Verification of *L. plantarum* and *S. cerevisiae* Titration Measurement

To validate the ability of the image cytometer to measure the titration of yeast and bacteria monocultures, we prepared a monoculture of *S. cerevisiae* at dilution fractions of 0.1, 0.3, 0.5, 0.7, 0.9, and 1.0, as well as a monoculture of *L. plantarum* at dilution fractions of 0.1, 0.25, 0.50, 0.75, and 1.00. After staining the samples with AO/PI and SYTO BC, respectively, they were counted with the image cytometer to generate the titration results.

**Fig. 2.**
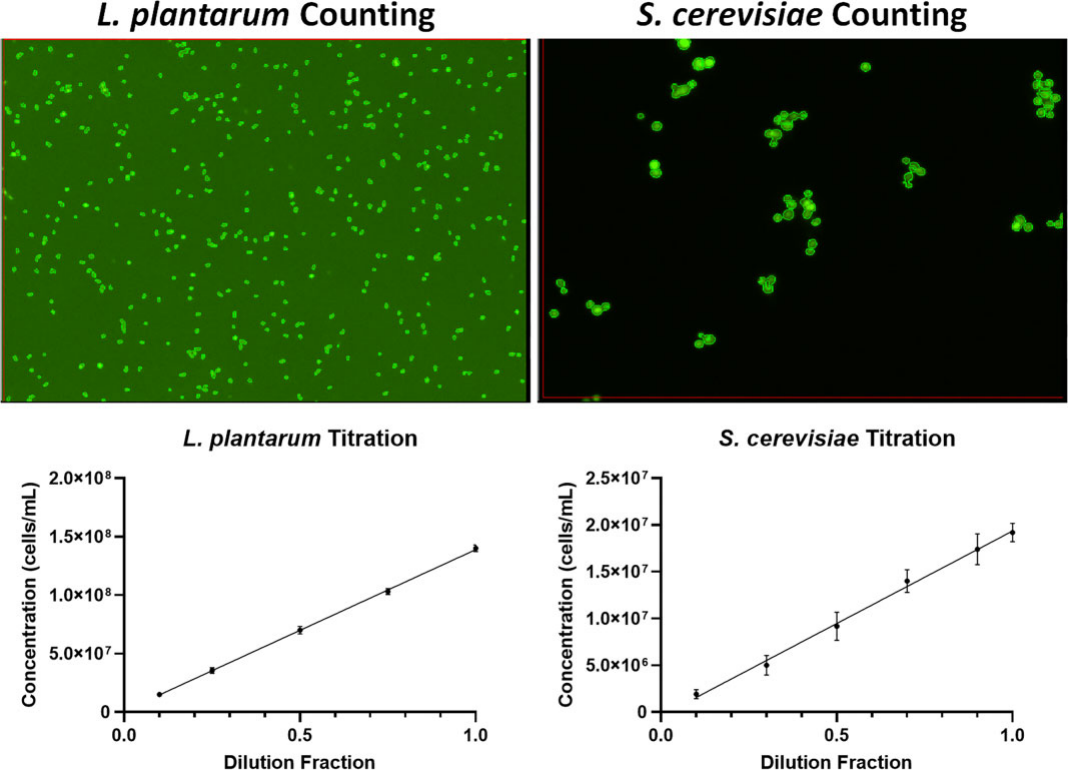
(Top) Counted fluorescent images of *Lactobacillus plantarum* and *Saccharomyces cerevisiae* and (Bottom) monoculture titration concentration measurement results.

The counted fluorescent images and results for *L. plantarum* and *S. cerevisiae* are shown in Fig. 2 (top), which demonstrated the direct counting of fluorescently stained yeast and bacteria. The titration results showed a highly linear response for both microorganisms with *R*^2^ values of 0.997 and 0.967 for *L. plantarum* and *S. cerevisiae*, respectively (bottom). This experiment repeated previously demonstrated cell counting methods using AO/PI and SYTO BC staining (Hodgkin et al., 2020; Saldi et al., 2014). The green outlines in the images showed individual yeast and bacteria counted. Furthermore, the ANOVA analysis showed that image cytometry and manual counting were statistically comparable when counting the monocultures of *L. plantarum* and *S. cerevisiae* for the three repeated experiments with a *p*-value of .49 and .85, respectively (Table 1).

**Table 1.** ANOVA comparison analysis between manual counting and image cytometry for *Lactobacillus plantarum* and *Saccharomyces cerevisiae* monoculture titration experiments

*L. plantarum* titration				
Trial	Manual counting Log(CFU/mL)	Image cytometry Log(cells/mL)	Difference	*p*-value
**1**	9.65 ± 0.04	9.71 ± 0.25	−0.06	.49
**2**	9.65 ± 0.06	9.74 ± 0.26	−0.09	
**3**	9.80 ± 0.04	9.77 ± 0.19	0.03	
***S. cerevisiae* titration**				
**1**	7.66 ± 0.07	7.54 ± 0.17	0.12	.85
**2**	7.32 ± 0.12	7.31 ± 0.08	0.01	
**3**	7.53 ± 0.08	7.58 ± 0.08	−0.05	

### Validation of *L. plantarum* and *S. cerevisiae* Mixture Measurement

To demonstrate the ability of the image cytometer to simultaneously count yeast and bacteria in mixed culture, we prepared three different samples with various ratios of *L. plantarum* and *S. cerevisiae* in the mixture. The purpose of the first two experiments was to show the simultaneous counting of *L. plantarum* and *S. cerevisiae* when keeping one microorganism constant and diluting the other. The third experiment was to show simultaneous counting when both microorganisms were diluted.

**Fig. 3.**
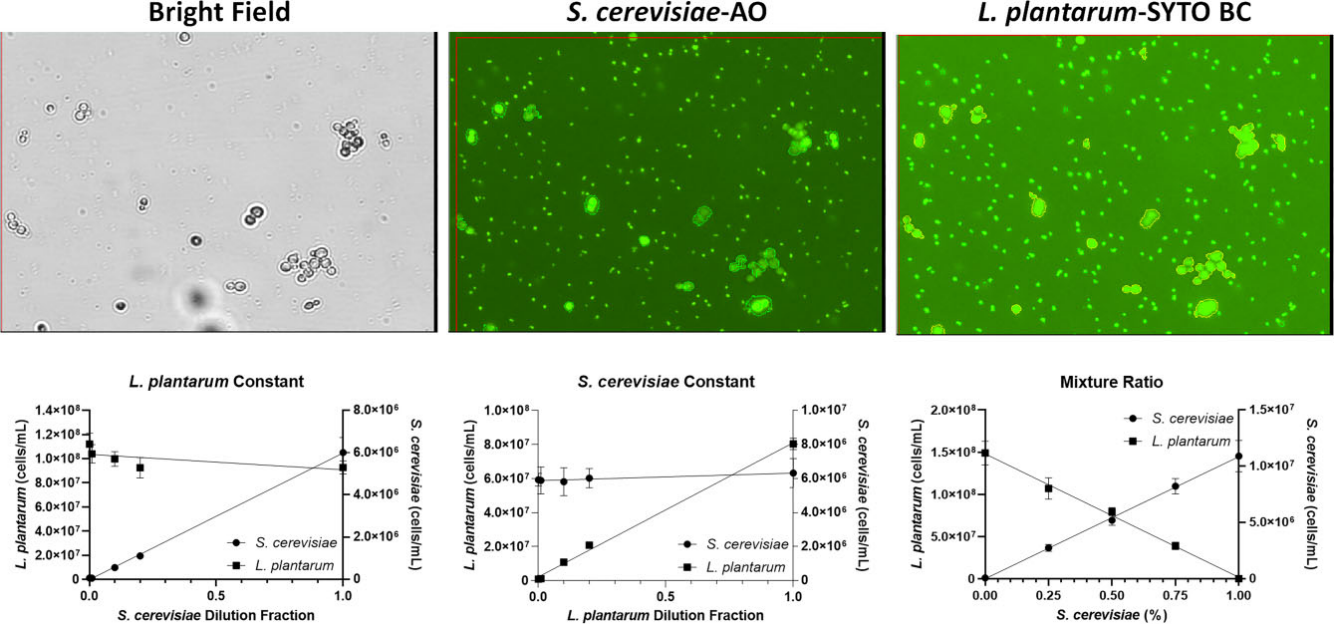
(Top) Bright field and counted fluorescent images of *Lactobacillus plantarum* and *Saccharomyces cerevisiae* and (Bottom) *L. plantarum* constant, *S. cerevisiae* constant, and mixture ratio concentration measurement results.

The bright field and fluorescent images, as well as the results of the mixed culture enumeration by image cytometry, are shown in Fig. 3, where the acquisition of both fluorescent channels was set to green to detect AO and SYTO BC. The parameters were set up to only count the yeast and bacteria in channels 1 and 2, respectively. The first experiment (*L. plantarum* constant) showed *R*^2^ values of 0.981 and 0.219 for yeast and bacteria, respectively. The second experiment (*S. cerevisiae* constant) showed *R*^2^ values of 0.056 and 0.994 for yeast and bacteria, respectively. The results indicated that the image cytometer was able to successfully measure the titration of yeast and bacteria, as well as measuring the constant concentrations. For the third experiment, the *R*^2^ values were 0.967 and 0.970 for yeast and bacteria, respectively, which showed that the image cytometer can measure titrations from both organisms simultaneously. The ANOVA analysis showed that image cytometry and manual counting were statistically comparable for all three different mixture experiments at *n* = 3 (Tables 2 and 3). First, keeping *L. plantarum* constant, the *p*-values were .58 and 0.50 for bacteria and yeast, respectively. Second, keeping *S. cerevisiae* constant, the *p*-values were .96 and 0.46, respectively. Finally, the bacteria and yeast ratio mixture experiment showed *p*-values at .18 and .08, respectively.

**Table 2.** ANOVA comparison analysis between manual counting and image cytometry for *Lactobacillus plantarum* and *Saccharomyces cerevisiae* constant and titration experiments

*S. cerevisiae* constant (*L. plantarum*)					*S. cerevisiae* constant (*S. cerevisiae*)				
Trial	Manual counting Log(CFU/mL)	Image cytometry Log(cells/mL)	Difference	*p*-value	Trial	Manual counting Log(CFU/mL)	Image cytometry Log(cells/mL)	Difference	*p*-value
**1**	9.68 ± 0.04	9.85 ± 0.07	−0.17	.58	**1**	7.66 ± 0.07	7.73 ± 0.08	−0.07	.50
**2**	9.89 ± 0.07	9.72 ± 0.04	0.32		**2**	7.57 ± 0.13	7.71 ± 0.07	−0.14	
**3**	9.79 ± 0.07	9.63 ± 0.08	0.16		**3**	7.50 ± 0.05	7.49 ± 0.13	0.07	
***L. plantarum* constant (*L. plantarum*)**					***L. plantarum* constant (*S. cerevisiae*)**				
**1**	9.69 ± 0.03	9.78 ± 0.03	−0.09	.96	**1**	7.66 ± 0.07	7.87 ± 0.05	−0.11	.46
**2**	9.49 ± 0.11	9.65 ± 0.03	−0.16		**2**	7.62 ± 0.11	7.62 ± 0.14	0.00	
**3**	9.76 ± 0.07	9.49 ± 0.05	0.26		**3**	7.47 ± 0.05	7.54 ± 0.06	−0.10	

**Table 3.** ANOVA comparison analysis between manual counting and image cytometry for *Lactobacillus plantarum* and *Saccharomyces cerevisiae* mixture ratio experiments

*L. plantarum* mixture ratio				
Trial	Manual counting Log(CFU/mL)	Image cytometry Log(cells/mL)	Difference	*p*-value
**1**	9.32 ± 0.06	9.68 ± 0.07	−0.36	.18
**2**	9.60 ± 0.12	9.76 ± 0.14	−0.16	
**3**	9.68 ± 0.08	9.65 ± 0.14	0.03	
***S. cerevisiae* mixture ratio**				
**1**	7.17 ± 0.06	7.67 ± 0.10	−0.50	.08
**2**	7.33 ± 0.12	7.74 ± 0.08	−0.41	
**3**	7.54 ± 0.15	7.51 ± 0.11	0.03	

It is important to note that fluorescent staining is critical for the proposed image cytometry method to distinguish between yeast and bacteria cells. Fluorescence-based image analysis can also minimize background noise and debris in bright-field imaging.

### Verification of *L. plantarum* and *S. cerevisiae* Measurement in Fermentation

The fermentation trials focused on producing a Berliner Wiesse product, a low-alcohol German sour beer that dates back to the 16th century. It is traditionally fermented with *S. cerevisiae* or *Brettanomyces* in combination with LABs using wort made from mixed extracted wheat and grain malt. In addition, Berliner Wiesse is traditionally brewed with small quantities of hops or without hops because the LABs are sensitive to compounds found in hops (Schurr et al., 2015) due to the antimicrobial properties that can disrupt cell membranes. Therefore, hops were removed from the fermentation recipe. The simplicity of the fermentation process aligned closely with the mixed-culture cell counting method developed in this work.

**Fig. 4.**
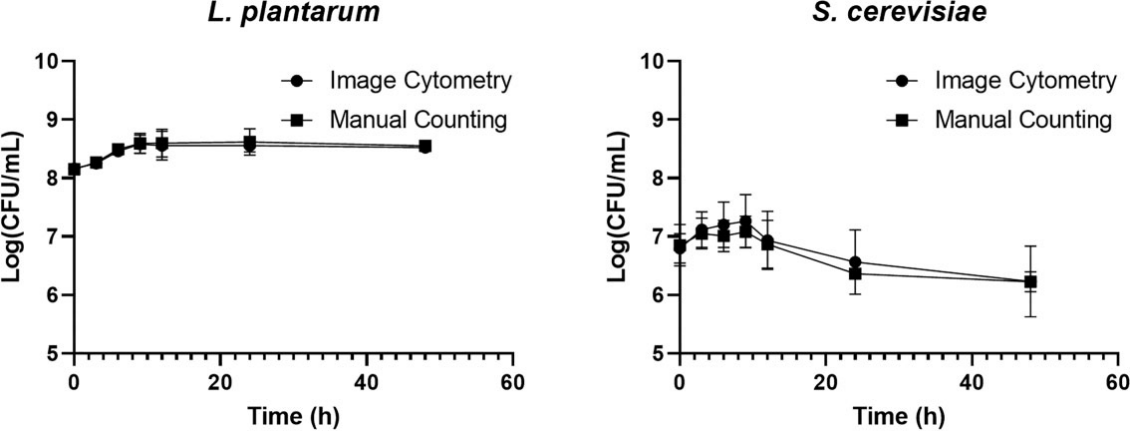
Time-course monitoring of *Lactobacillus plantarum* and *Saccharomyces cerevisiae* concentrations comparison between image cytometry and manual counting during the Berliner Weisse fermentation process.

The validated image cytometry cell counting method was used to determine the concentration of *S. cerevisiae* and *L. plantarum* during fermentation. The results showed a range of 5.93–7.38 log and 8.88–8.12 log, respectively (Fig. 4). The *S. cerevisiae* concentration increased in the first 9 hr of the fermentation and peaked at ∼1.82 × 10^7^ CFU/mL for both image cytometry and manual counting (APDA). The concentration decreased from 12 to 24 hr resulting in an average concentration of 3.72 × 10^6^ CFU/mL for image cytometry and 2.29 × 10^6^ for manual counting. Finally, the yeast concentration tapered off at ∼3.02 × 10^6^ cells/mL. *Saccharomyces cerevisiae* experienced the highest growth in the first 9 hr, followed by a slight reduction at every time point throughout the 48 hr, which could be caused by the lower pH (∼5.1) inhibiting the yeast replication or by the ending of primary fermentation (Bamforth, 2009). Some brewers may define the end of the primary fermentation when the SG falls below 1.030. Even at a low pH, if fermentable sugars are present, yeast will continue to lower the SG as they can better tolerate a low pH and increase alcohol concentration. This can result in some sour beer fermentation spanning many months, which is usually caused by the presence of *Brettanomyces*. Due to time constraints in this work, fermentation trials were monitored for 48 hr, when the SG fell below 1.030, indicating the completion of primary fermentation.

On the other hand, the *L. plantarum* concentration increased in the first 12 hr and then plateaued from 24 to 48 hr. The *L. plantarum* started with a concentration of 1.48 × 10^8^ CFU/mL for image cytometry and 1.41 × 10^8^ for manual counting (MRS). The concentration increased to ∼3.55 × 10^8^ and 3.98 × 10^8^ cells/mL for image cytometry and manual counting, respectively. The final concentration after 48 hr was ∼3.31 × 10^8^ and 3.55 × 10^8^ CFU/mL for image cytometry and manual counting, respectively. *Lactobacillus plantarum* was in a growth phase in the first 12 hr. Subsequently, *L. plantarum* plateaus, and the increases were negligible until the 48-hr time point. This fermentation characteristic was observed in each of the three fermentation trials. Lactic acid bacteria are the most active in the first 12 hr of fermentation, where a steep reduction in the pH can be seen in the fermenting wort for sour beers. In general, the pH can reduce from mid-5 to low 3, where the LABs are inhibited at low pH and can no longer replicate. The plateauing of concentration corresponded with the reduction of LAB activity that occurred after 12 hr.

The calculated ANOVA comparison between image cytometry and manual counting showed statistically comparable results between the two counting methods as well as the two microorganisms during the fermentation (Table 4). The *p*-values calculated for bacteria and yeast were .73 and .81, respectively, which indicates that there was no significant difference between the novel image cytometry method and traditional manual counting.

**Table 4.** ANOVA comparison analysis between manual counting and image cytometry for *Lactobacillus plantarum* and *Saccharomyces cerevisiae* mixed culture Berliner Wiesse fermentation

*L. plantarum* mixed culture fermentation				
Trial	Manual counting Log(CFU/mL)	Image cytometry Log(cells/mL)	Difference	*p*-value
**1**	8.51 ± 0.25	8.51 ± 0.22	0.00	.81
**2**	8.38 ± 0.12	8.40 ± 0.12	−0.02	
**3**	8.40 ± 0.12	8.42 ± 0.17	−0.02	
***S. cerevisiae* mixed culture fermentation**				
**1**	6.72 ± 0.47	6.43 ± 0.43	0.29	.73
**2**	6.98 ± 0.40	7.20 ± 0.41	−0.22	
**3**	6.64 ± 0.26	6.99 ± 0.21	−0.35	

The results from the mixed culture fermentation experiment were used to demonstrate the ability of the novel image cytometry method to simultaneously count yeast and bacteria directly from fermentation samples while simulating standard industry practices. We found no significant differences between the counting methods. Therefore, the proposed image cytometry method may be utilized for commercial brewing applications.

## Conclusion

In this work, we have demonstrated the capability of the Cellometer X2 image cytometer to automatically distinguish and count *L. plantarum* and *S. cerevisiae* in monoculture, different mixture ratios, and mixed culture fermentation. The novel method was validated by comparing with traditional CFU manual counting, which demonstrated highly comparable cell concentration results for all experiments. The proposed image cytometry method in combination with fluorescent stains and size exclusion image analysis algorithms is the only cell counting technique that can count *L. plantarum* and *S. cerevisiae* simultaneously in a mixed culture. Therefore, it can provide an effective and efficient tool to characterize mixed cultures during fermentation, producing more consistent and higher-quality beverage products. Further research will be performed to expand the species of bacteria and *Brettanomyces* yeast and to assess the utility of the method in other fermented beverages.

## References

[bib1] Bamforth C. (2009). Beer: tap into the art and science of brewing (3rd ed.). Oxford University Press.

[bib2] Boulton C., Quain D. (2006). The brewing process. In Boulton C., Quain D. (Eds). Brewing yeast and fermentation. Blackwell Science Ltd.

[bib3] Bourdichon F., Casaregola S., Farrokh C., Frisvad J. C., Gerds M. L., Hammes W. P., Harnett J., Huys G., Laulund S., Ouwehand A., Powell I. B., Prajapati J. B., Seto Y., Schure E. T., Boven A. V., Vankerckhoven V., Zgoda A., Tuijtelaars S., Hansen E. B. (2012). Food fermentations: microorganisms with technological beneficial use. International Journal of Food Microbiology, 154(3), 87–97. 22257932 10.1016/j.ijfoodmicro.2011.12.030

[bib4] Brewers Association Releases Annual Craft Brewing Industry Production Report for 2020 . (2021). https://www.brewersassociation.org/press-releases/2020-craft-brewing-industry-production-report/, accessed April 6, 2021.

[bib5] Cebeci A., Gürakan C. (2003). Properties of potential probiotic *Lactobacillus plantarum* strains. Food Microbiology, 20(5), 511–518.

[bib6] Chan L. L., Kury A., Wilkinson A., Berkes C., Pirani A. (2012). Novel image cytometric method for detection of physiological and metabolic changes in *Saccharomyces cerevisiae*. Journal of Industrial Microbiology and Biotechnology, 39(11), 1615–1623. 10.1007/s10295-012-1177-y 22864608

[bib7] Chan L. L., Lyettefi E. J., Pirani A., Smith T., Qiu J., Lin B. (2011). Direct concentration and viability measurement of yeast in corn mash using a novel imaging cytometry method. Journal of Industrial Microbiology & Biotechnology, 38(8), 1109–1115. 10.1007/s10295-010-0890-7 20960026

[bib8] Hesseltine C. W. (1992a). Food fermentations: mucorales in ragi and related products. In Leatham G. F. (Ed.), Frontiers in industrial mycology. Springer.

[bib9] Hesseltine C. W. (1992b). Mixed-culture fermentations. In Applications of biotechnology in traditional fermented foods. National Academies Press.

[bib10] Hodgkin M., Purseglove S. M., Chan L. L.-Y., Perry J., Bolton J. (2020). A novel image cytometry-based *Lactobacillus* bacterial enumeration method for the production of kettle sour beer. Journal of Microbiological Methods, 177, 106031. 32805365 10.1016/j.mimet.2020.106031

[bib11] Liszkowska W., Berlowska J. (2021). Yeast fermentation at low temperatures: adaptation to changing environmental conditions and formation of volatile compounds. Molecules, 26(4), 1–20. 10.3390/molecules26041035 PMC7919833 33669237

[bib12] Martyniak B., Bolton J., Kuksin D., Shahin S. M., Chan L. L.-Y. (2017). A novel concentration and viability detection method for Brettanomyces using the Cellometer image cytometry. Journal of Industrial Microbiology & Biotechnology, 44(1), 119–128. 27838895 10.1007/s10295-016-1861-4

[bib13] Mathur H., Beresford T. P., Cotter P. D. (2020). Health benefits of lactic acid bacteria (LAB) fermentates. Nutrients, 12(6), 1679. 32512787 10.3390/nu12061679 PMC7352953

[bib14] Prajapati J. B., Nair B. M. (2003). The history of fermented foods. In Farnworth E. R. (Ed.), Fermented functional foods (pp. 1–25). CRC Press.

[bib15] Rojas A. A. R., Swidah R., Schindler D. (2022). Microbes of traditional fermentation processes as synthetic biology chassis to tackle future food challenges. Frontiers in Bioengineering and Biotechnology, 10, 982975. 10.3389/fbioe.2022.982975 36185425 PMC9523148

[bib16] Saldi S., Driscoll D., Kuksin D., Chan L. L.-Y. (2014). Image-based cytometric analysis of fluorescent viability and vitality staining methods for ale and lager fermentation yeast. Journal of American Society of Brewing Chemists, 72(4), 253–260.

[bib17] Sanders E. R. (2012). Aseptic laboratory techniques: plating methods. Journal of Visualized Experiments, 63, 3064. 10.3791/3064 PMC4846335 22617405

[bib18] Schurr B. C., Hahne H., Kuster B., Behr J., Vogel R. F. (2015). Molecular mechanisms behind the antimicrobial activity of hop iso-α-acids in *Lactobacillus brevis*. Food Microbiology, 46, 553–563. 25475328 10.1016/j.fm.2014.09.017

[bib19] Smid E. J., Lacroix C. (2013). Microbe–microbe interactions in mixed culture food fermentations. Current Opinion in Biotechnology, 24(2), 148–154. 23228389 10.1016/j.copbio.2012.11.007

[bib20] Soundharrajan I., Kim D., Kuppusamy P., Muthusamy K., Lee H. J., Choi K. C. (2019). Probiotic and *Triticale* silage fermentation potential of *Pediococcus pentosaceus* and *Lactobacillus brevis* and their impacts on pathogenic bacteria. Microorganisms, 7(318), 1–19. 10.3390/microorganisms7090318 PMC6780645 31487912

[bib21] Spencer J. F. T., Spencer D. M. (1997). Ecology: where yeasts live. In Spencer J. F. T., Spencer D. M. (Eds.), Yeasts in natural and artificial habitats (pp. 33–58). Springer.

[bib22] Statista . (2022). Non-alcoholic beverages and soft drinks in the U.S.––statistics & facts. Statista. https://www.statista.com/topics/1662/non-alcoholic-beverages-and-soft-drinks-in-the-us/, accessed Decembe 6, 2022

[bib23] Swiegers J. H., Bartowsky E. J., Henschke P. A., Pretorius I. S. (2005). Yeast and bacterial modulation of wine aroma and flavour. Australian Journal of Grape and Wine Research, 11(2), 139–173.

[bib24] Thomas P., Sekhar A. C., Mujawar M. M. (2012). Nonrecovery of varying proportions of viable bacteria during spread plating governed by the extent of spreader usage and proposal for an alternate spotting-spreading approach to maximize the CFU. Applied Microbiology, 113(2), 339–350. 10.1111/j.1365-2672.2012.05327.x 22563785

[bib25] Tonsmeire M. (2014). American sour beers: innovative techniques for mixed fermentations. Brewers Publications.

